# The German National Cohort: Ophthalmic Assessment, Baseline Profile, and Potential for Artificial Intelligence–Based Eye Research

**DOI:** 10.1016/j.xops.2026.101408

**Published:** 2026-09-11

**Authors:** Camila Roa, Ansgar Beuse, Alexandra Schweig, Sarah Müller, Klaus Berger, Caroline Brandl, Titus Brinker, Anne Elbrecht, Robert Finger, Gerd Geerling, Karin Halina Greiser, Carsten Grohmann, Kathrin Günther, Iris Heid, André Karch, Thomas Keil, Jessica Krepel, Michael Leitzmann, Claudia Meinke-Franze, Annette Peters, Sabine Schipf, Matthias Schulze, Alexander K. Schuster, Stefan N. Willich, Martin A. Leitritz, Marius Ueffing, Philipp Berens

**Affiliations:** 1 Hertie Institute for AI in Brain Health, University of Tübingen, Tübingen, Germany; 2 Tübingen AI Center, University of Tübingen, Tübingen, Germany; 3 Department of Ophthalmology, University Medical Center Hamburg-Eppendorf (UKE), Hamburg, Germany; 4 University Eye Hospital, Centre for Ophthalmology, University of Tübingen, Tübingen, Germany; 5 Institute of Epidemiology and Social Medicine, University of Münster, Münster, Germany; 6 Department of Genetic Epidemiology, University of Regensburg, Regensburg, Germany; 7 Department of Ophthalmology, University of Regensburg, Regensburg, Germany; 8 German Cancer Research Center (DKFZ) Heidelberg, Division of Cancer Epidemiology, Heidelberg, Germany; 9 Department of Epidemiological Methods and Etiological Research, Leibniz Institute for Prevention Research and Epidemiology – BIPS, Bremen, Germany; 10 Department of Ophthalmology, Medical Faculty Mannheim, University of Heidelberg, Mannheim, Germany; 11 Department of Ophthalmology, Heinrich-Heine University, Düsseldorf, Germany; 12 Institute of Social Medicine, Epidemiology and Health Economics, Charité - Universitätsmedizin Berlin, Berlin, Germany; 13 Institute of Clinical Epidemiology and Biometry, University of Würzburg, Würzburg, Germany; 14 State Institute of Health I, Bavarian Health and Food Safety Authority, Erlangen, Germany; 15 Helmholtz Center for Infection Research, Braunschweig, Germany; 16 Institute for Epidemiology and Prevention Medicine, University of Regensburg, Regensburg, Germany; 17 Institut für Community Medicine, Universitätsmedizin Greifswald, Greifswald, Germany; 18 Chair of Epidemiology, IBE, Faculty of Medicine, LMU Munich, Germany; 19 Helmholtz Zentrum München, German Research Center for Environmental Health (GmbH), Institute of Epidemiology, Neuherberg, Germany; 20 Department of Molecular Epidemiology, German Institute of Human Nutrition Potsdam-Rehbruecke, Nuthetal, Germany; 21 Institute of Nutritional Science, University of Potsdam, Nuthetal, Germany; 22 Department of Ophthalmology, University Medical Center of the Johannes Gutenberg-University Mainz, Mainz, Germany; 23 Institute for Ophthalmic Research, Centre for Ophthalmology, University of Tübingen, Tübingen, Germany

**Keywords:** Artificial intelligence, Epidemiology, Prospective cohort study, Fundus image, Quality control

## Abstract

**Objective:**

To describe the ophthalmic examination protocol within the German National Cohort (NAKO)/NAKO Gesundheitsstudie, to report the baseline profile of participants undergoing ophthalmic assessment, and to illustrate the potential as a population-based resource for artificial intelligence (AI) research in eye health.

**Design:**

Baseline analysis of ophthalmic data within the nationwide, population-based multicenter prospective NAKO study.

**Participants:**

Forty-eight thousand four hundred and sixty adults in the ophthalmic level 2 module of 205 053 adults enrolled in NAKO, aged 19–74 years, with a mean age 48.9 ± 12.5 years, and 52.7% were male.

**Methods:**

All participants underwent standardized biomedical examinations and detailed questionnaire-based data collection, including nondilated color fundus imaging, visual acuity testing, and a brief ocular history. Ocular health was summarized using descriptive statistics. Fundus image quality and morphological features (e.g., cup-to-disc ratio and arteriolar-to-venular ratio) were assessed using open-source deep learning models. Standard deep learning architectures were trained on the fundus images to predict age, sex, and blood pressure.

**Main Outcome Measures:**

Percentage of fundus images graded as good quality; mean absolute error (MAE) for age and blood pressure prediction; accuracy for sex prediction.

**Results:**

The analysis includes 48 460 participants who completed the ophthalmic baseline examination of NAKO. Mean visual acuity (logarithm of the minimum angle of resolution) was 0.01 ± 0.20 (left eye, mean ± standard deviation) and 0.03 ± 0.21 (right eye). Self-reported ocular disease prevalence was 4.2% for cataract, 2.0% for glaucoma, and 0.9% for macular degeneration; 68.2% of fundus images were classified as gradable using 4 deep learning–based quality grading models. Morphological features showed systematic differences across age groups. Standard deep learning architectures showed comparative performance to the state-of-the-art for age, sex, and blood pressure prediction (2.96 MAE for age prediction, 0.84 accuracy for sex prediction, and 10.78 and 6.97 MAE for systolic and diastolic blood pressure prediction).

**Conclusions:**

The German National Cohort provides a large-scale, nationwide population-based resource with visual acuity measurements and systemic health indicators, as well as color fundus images in about 50 000 NAKO participants. These data lay the groundwork for studying eye health in the general adult population in Germany and can serve as a strong foundation for developing and validating AI tools in eye health research.

**Financial Disclosure(s):**

Proprietary or commercial disclosure may be found in the Footnotes and Disclosures at the end of this article.

The German National Cohort (NAKO)/NAKO Gesundheitsstudie is a nationwide, population-based prospective study designed to investigate the determinants, risk factors, early markers, trajectories, and long-term consequences of major chronic diseases in Germany.^1^^,^^2^ With 205 053 participants enrolled at baseline across 18 study centers, NAKO is one of the largest deeply phenotyped cohorts worldwide. It is among large population-based studies with >100 000 participants, including nationwide studies such as the UK Biobank (UKB)^3^ and the China Kadoorie Biobank.^4^ The German National Cohort’s comprehensive design incorporates multimodal imaging, biospecimen collection, environmental and behavioral profiling, and a planned overall duration of 25 to 30 years.^1^^,^^5^ A subgroup of >55 000 participants was offered an extended examination program (so called level 2 examinations).

Within this framework, the ophthalmology module was established as one of the level 2 investigations, designed to serve 3 purposes that define its intended scope of use. First, NAKO provides population-based reference data on visual acuity and self-reported ocular disease prevalence in the German adult population, enabling epidemiological studies of eye health across age, sex, and region, and—as follow-up data accumulate—longitudinal assessment of visual decline and its determinants. Second, the standardized acquisition of color fundus images across 18 study centers creates a large-scale resource for the development, training, and external validation of deep learning models for automated retinal phenotyping and oculomics research, linking retinal features to the extensive systemic health data collected in the NAKO. Third, the combination of imaging, visual function, and self-reported ocular disease in a deeply phenotyped cohort with a planned follow-up of 25–30 years provides a foundation for investigating associations between ocular measures and nonocular conditions, such as diabetes, hypertension, and cardiovascular disease—in both directions.

In this paper, we describe the examination protocol and quality assurance framework of the NAKO ophthalmology module, report the baseline profile of the 48 460 participants who underwent ophthalmic assessment, and demonstrate the suitability of the data for artificial intelligence (AI)–based research by evaluating fundus image quality with open-source deep learning models, extracting morphological features, and training standard architectures to predict age, sex, and blood pressure. This paper serves as the primary methodological reference for the ophthalmic assessment within the NAKO. Researchers using NAKO ophthalmic data—including fundus images, visual acuity measurements, derived quality gradings, morphological features, or trained models—are asked to cite this paper alongside the general NAKO study reference.^1^

## Methods

### Design, Baseline Recruitment, and Ethical Approval

The NAKO is a large, prospective, population-based cohort study conducted in Germany. Participants were randomly selected from compulsory population registries in 16 German regions using age- and sex-stratified sampling. They were examined between 2014 and 2019 in 18 study centers representing urban, suburban, and rural settings.^6^ The NAKO aimed to recruit approximately 200 000 residents between the ages of 20 and 69.^5^

The baseline recruitment yielded 205 415 participants aged 19–74 years. The inclusion criteria were: residing in the study catchment area, being within the target age range at the time of invitation, ability to provide written informed consent, and proficiency in the German language to understand questionnaires and instructions. The exclusion criteria included being unable to consent. The overall response rate averaged 15.6%, with higher participation among older individuals and women with differences across regions.^1^^,^^6^ Recruitment was monitored to ensure representativeness across predefined strata. After 362 participants withdrew informed consent, the sample consisted of 205 053 individuals as of October 2019.^1^

Baseline assessment included standardized, computer-assisted face-to-face interviews, self-administered questionnaires, standardized physical and medical examinations, and biosample collection. A core set of questionnaires and examinations was offered to all participants (so called level 1 modules), and an extended set of in-depth examinations and questionnaire modules (level 2 modules) was offered to a 20% random sample of all participants plus to additional participants undergoing magnetic resonance imaging at 5 of the 18 study centers. Information about the items in the questionnaire is available as part of the NAKO Data Handbook^1^. For training of the AI-based models regarding estimation of age, sex, and blood pressure, we used data obtained during interview and during measurement of the sitting blood pressure with the OMRON-HEM 705 IT.^1^

The study protocol of NAKO was approved by the ethics committees of all participating institutions, and all participants provided written informed consent in accordance with German legal and data protection requirements. It is conducted in accordance with the Declaration of Helsinki and national standards for good clinical and epidemiological practice.^1^

### The Ophthalmology Module

The intensified level 2 examination of the NAKO includes an ophthalmology module comprising visual acuity testing, nonmydriatic color fundus imaging, and a standardized self-report questionnaire on ocular disease history. The module was conducted in 56 971 participants of the baseline examination (Fig 1). Of those, 48 460 completed at least one visual acuity measurement and one fundus image for ≥1 eye, according to the data set provided by the TransferHub (Fig 1, Table 1). The complete eye examination required approximately 10 minutes.

**Figure 1:**
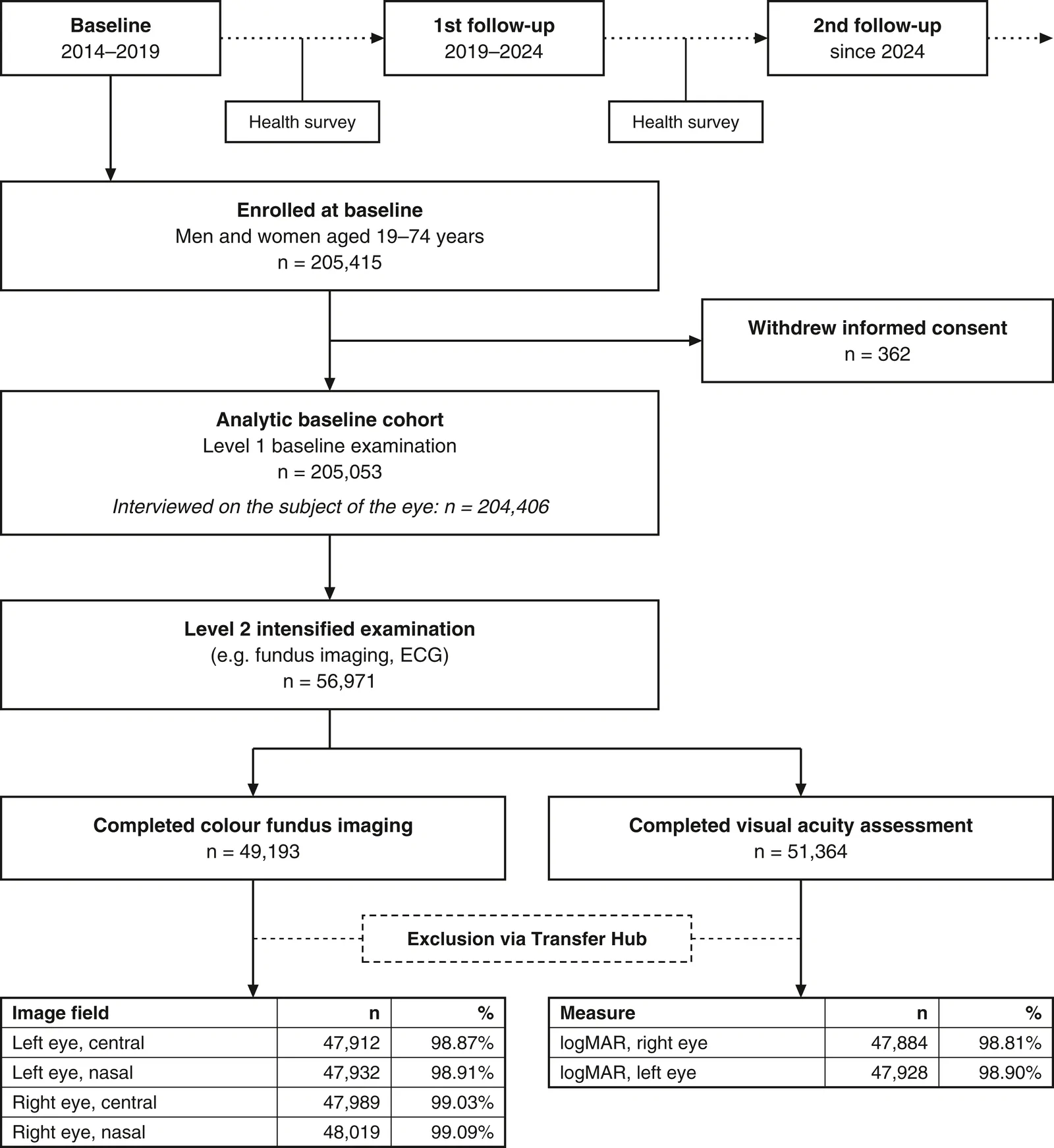
Flowchart of study participants for the baseline examination in the level 2 ophthalmology submodule of the NAKO. Numbers are reported as in n = participants. ECG = electrocardiography; logMAR = logarithm of the minimum angle of resolution; NAKO = German National Cohort.

**Table 1:** Summary of Demographic, Systemic, and Eye Health Variables of n = 48 460 Participants Provided by NAKO TransferHub from the Baseline in the NAKO Ophthalmology Data Set

Variable	Total (n = 48 460)
Continuous	mean ± SD [min, max]
Age (years)	48.91 ± 12.53 [19, 74]
Systolic blood pressure (mmHg)	127.14 ± 16.16 [75, 236]
Diastolic blood pressure (mmHg)	78.39 ± 9.96 [44, 136]
Left eye logMAR	0.01 ± 0.20 [–0.20, 1.20]
Right eye logMAR	0.03 ± 0.21 [–0.20, 1.20]
Categorical	n (%)
Sex	
Male	25 534 (52.69%)
Female	22 926 (47.31%)
Smoking status	
Not a smoker	22 836 (47.12%)
Former smoker	15 303 (31.58%)
Smoker	9185 (18.95%)
Missing	1136 (2.34%)
High blood pressure (yes)	11 566 (23.87%)
Diabetes mellitus (yes)	2380 (4.91%)
Cataracts (yes)	2042 (4.21%)
Glaucoma (yes)	971 (2.00%)
Macular degeneration (yes)	429 (0.89%)

logMAR = logarithm of the minimum angle of resolution; NAKO = German National Cohort; SD = standard deviation.

Continuous variables are reported as mean ± SD [min, max], and discrete variables as n (%). Note: Percentages are relative to the total number of participants. Missing values for disease variables were high blood pressure, 42 (0.09%); diabetes mellitus, 64 (0.13%); cataracts, 121 (0.25%); glaucoma, 151 (0.31%); macular degeneration, 161 (0.33%).

#### Visual Acuity Assessment

Visual acuity was tested monocularly while participants wore their walk-in distance correction (glasses or contact lenses). An additional occluder fully covered the nontested eye and incorporated a central aperture in front of the eye under examination. If visual acuity exceeded a logarithm of the minimum angle of resolution value of 0.0, a rotatable insert with multiple 2.0 mm perforated openings was additionally positioned in front of this aperture to enhance letter recognition and mitigate residual refractive errors. Illuminated optotype charts with Latin letters, based on the ETDRS protocol, were used at a testing distance of 4 m. In case of room length restrictions, a fixed mirror was used to achieve the required distance. Visual acuity was assessable from –0.20 to 1.20. The test was started by reading all letters from the top of the letter chart from left to right. It was terminated when <3 of 5 letters on the same line were correctly identified. Results—including the type of refractive correction, identified optotypes, and notable observations—were digitally documented by study staff.^7^ The visual acuity protocol was harmonized with and adapted from the UKB examination protocol to permit future joint analyses.

#### Fundus Imaging

Retinal imaging was performed without mydriasis using a DRS fundus camera (Revenio group, icare, Finland; formerly CenterVue S.p.A.). The camera has a field of view of 45° × 40°, an autofocus range of –15 to +15 D, internal fixation light-emitting diodes, a working distance of 37 mm, and a 5-megapixel sensor (2592 × 1944 pixels), yielding a resolution of 48 pixels/degree and a retinal resolution of 15 μm^2^ for the standard eye. For each eye, one macula-centered and one optic disc–centered image was acquired. If the first image was judged to be of insufficient quality, the study staff repeated the acquisition; all images—including retakes—were saved. Fundus imaging was performed in a darkened room and caused only minor inconvenience (brief glare) for the participants.

#### Ocular Disease History

Alongside the eye examination, participants were assessed for self-reported macular degeneration, cataract, optic nerve inflammation, and glaucoma using a standardized questionnaire.

#### Exclusion Criteria

The exclusion criteria for visual acuity testing and fundus imaging were assessed prior to testing and included: participant refusal, severe ocular infection, ocular surgery within the prior 4 weeks, prosthetic eye, and known blindness. Fundus imaging was additionally contraindicated in participants with photosensitive epilepsy. Testing could be discontinued at any time by the participant, examiner, or due to technical issues (e.g., camera malfunction).

### Quality Assurance and Organizational Oversight

The ophthalmology module was developed by a panel of ophthalmic, epidemiological, and information-technology experts from Germany (NAKO eye expert group), which is responsible for the development of the ophthalmology program, oversight, and support of future project development. A pretest study was conducted to test standard operating procedures for visual acuity assessment and color fundus imaging as well as different fundus cameras conducted.^7^ Standard operating procedures were modified to maximize information content versus participants' time in the study center to ensure feasibility at the scale of NAKO.

All examination procedures followed standard operating procedures, and all study personnel were trained and certified before collecting data. The NAKO-retina competence unit is responsible for overall management and quality assurance of the eye data across all 18 study centers. This includes regular (remote) site visits to ensure appropriate and equal on-site conditions, training sessions to ensure the integrity and accuracy of examinations and data processing, assessment of incidental findings, and continuous quality assessment through ongoing communication with the central data collection units and study centers.

Following the approval for pseudonymized analysis, fundus images were transferred to the Reading Centre at the Department of Ophthalmology, University Hospital Tübingen, Germany. Manual grading and annotation of the images from a list of predefined findings was performed at the Reading Centre. These annotations are being made available through the NAKO TransferHub. The Reading Centre provides specialized personnel, state-of-the-art equipment, and expert knowledge to support computer-assisted, standardized evaluation of the data.

### Data Availability and Governance

All data described in this paper are accessible through the official NAKO TransferHub (https://transfer.nako.de). Access requires submission of a Use/Access Application, which is reviewed by the NAKO Use and Access Committee for scientific merit, feasibility, consistency with the study's objectives and participants' informed consent, and compliance with legal and ethical standards. Upon approval by the Board of Directors and conclusion of a Use/Access Agreement, applicants receive a time-limited, nonexclusive, nontransferable right to use the requested data in pseudonymized form for the approved purpose. Applicant institutions must be located within the European Economic Area or in a jurisdiction with adequate data protection safeguards. Commercial intentions must be disclosed and require separate approval. Users are required to submit results data and metadata to NAKO e.V. within 12 months of project completion and to delete all provided data upon expiry of the authorized period of use. Imaging data may only be used in publications in a manner that precludes reidentification of participants.

To protect participant privacy, the NAKO employs a multilevel pseudonymization architecture in accordance with the guidelines of the TMF (Technology, Methods, and Infrastructure for Networked Medical Research). Personally identifying data (e.g., name, address, and date of birth) are stored exclusively at the organizationally independent Trust Centre (Unabhängige Treuhandstelle) and are never accessible to researchers. Medical study data are stored and processed only under domain-specific pseudonyms—independently generated random identifiers that differ across data systems (e.g., study database, laboratory information system, and research database). For each approved research project, a project-specific analysis data set is compiled with a further layer of repseudonymization, taking into account the reidentification risk arising from the specific combination of variables provided. Due to this governance framework and the requirements of German data protection law, the data cannot be deposited in an unrestricted public repository. The Use and Access Policy, Data Protection and IT Security Concept, and ethics documentation are available at https://nako.de/en/research/transferhub/. A Healthsheet for this data set, following the framework of Rostamzadeh et al, is provided as Supplementary Material.

### Statistical Analyses

We performed a descriptive analysis of the study population, summarizing basic demographic information (sex, age, and study site), general health related variables (blood pressure, smoking status, and diabetes), and ophthalmic variables (visual acuity, presence of eye diseases in self-report). Continuous variables are presented as means and standard deviations as well as minimum and maximum values. Categorical variables were summarized using absolute counts and percentages. Age-group distributions were calculated separately for men and women and reported as proportions of the total sample. Missing data were reported explicitly and no imputation was performed. All analyses were performed in Python (Python Software Foundation) using standard statistical procedures.

### Automated Fundus Image Analysis

#### Preprocessing

We used the Fundus Image Toolbox (FIT)^8^ to crop and center the fundus images appropriately.

#### Automatic Assessment of Image Quality

To automatically assess the quality of the fundus images, we used 4 different open-source software packages that include quality prediction models. For all models, we used default parameters and applied them to the preprocessed fundus images resized to 512 × 512.

• Fundus Image Toolbox^8^: a python package for fundus image processing that includes quality grading, fovea and optic disc localization, vessel segmentation, registration, and circle cropping. Quality is rated on a scale from 0 to 1 (ungradable to gradable) by an ensemble of standard deep learning architectures (ResNets and EfficientNets) trained on the DeepDRiD and DrimDB data sets.^9^^,^^10^ We used the default threshold of 0.25, classifying images with a score of ≥0.25 as gradable.
• AutoMorph^11^: a pipeline for automated analysis of retinal images that includes preprocessing, quality grading, vessel, artery, vein, and disc segmentation and morphological feature measurement. For quality grading, a standard deep learning architecture (EfficientNet-B4) trained on the EyeQ data set^12^ classifies images as good, usable, or reject. We considered good and usable images as gradable.
• VascX^13^: a pipeline for fundus analysis that includes preprocessing, quality grading, vessel artery-vein and disc segmentation, and fovea localization. The quality grading model is an ensemble of fine-tuned deep learning models (ResNet101) on the EyeQ data set that classifies images as good, usable, or reject. We considered good and usable images as gradable.
• RetiRatey (https://github.com/justinengelmann/RetiRatey): a fine-tuned small convolutional variant of the MobileNet-V4 family that rates the quality of retinal images on a scale from 1 to 4 (1: Very good, 2: Good, 3: Borderline, and 4: Bad) for different regions of interest: the macula, the disc, and the vessels. We considered images with scores <3 on all 3 regions of interest as gradable.

We validated automatic grading procedures on a subset of n = 100 images of different quality sampled randomly. These images were graded by 5 expert annotators as excellent, good, fair, poor, and unusable. The average Cohen κ between raters was high on average, indicating high interrater agreement (κ = 0.73). The gradings of different annotators were aggregated by averaging and majority vote. Spearman ρ between gradings and continuous model outputs as well as Cohen between binarized gradings and model outputs was used to quantify association strength. Automatic gradings by all 4 methods will be made available via the NAKO TransferHub.

#### Automatic Extraction of Morphological Fundus Image Features and Epidemiological Analysis

We used the AutoMorph toolbox^11^ to extract morphological measurements from the macula-centered retinal images, using its built-in quality prediction module as part of the end-to-end pipeline, which was designed and tuned for morphological feature extraction. We focused on the following measurements: the vertical cup to disk ratio, along with the central retinal artery equivalent (CRAE), the arteriolar-to-venular ratio (AVR), and the vessel density of zone b.^14^ We did not correct for magnification due to differences in axial length, corneal curvature, or refractive error, as this information was not available in the NAKO module. For these variables, we analyzed their relationship with age and sex using generalized additive models via the R package mgcv (version 1.9-4), fitting a smooth term for age, which was allowed to differ between sexes, resulting in the formula y ∼ sex + s (age, by = sex), where y is the variable of interest.^15^ We removed outliers using the interquartile range (IQR) method, applied independently to each feature. Outliers below Q1 – 1.5×IQR or above Q3 + 1.5×IQR were excluded, where IQR = Q3 - Q1. Omitting outlier removal did not qualitatively change the results. Extracted morphological properties will be made available through the NAKO TransferHub.

#### Deep Learning–Based Prediction of Systemic Variables

We trained several standard deep learning models—ResNet18, ResNet50, Inceptionv3, Xception, EfficientNetv2-Small, ViT-Small, and Swin-Tiny^16^, ^17^, ^18^, ^19^, ^20^, ^21^—to predict age, blood pressure, and sex from fundus images from the NAKO baseline examination. The size of the transformer models was chosen to yield models of a size in the same order of magnitude as a ResNet50. Preliminary experiments revealed that ImageNet pretraining was helpful to obtain better performing models in less time. Thus, all models were initialized with ImageNet weights and fine-tuned for a maximum of 25 epochs with early stopping. We used a batch size of 256 and an AdamW optimizer with a learning rate of 10^-4^ and weight decay of 10^-4^. We trained the classifiers with cross-entropy loss and the regression models with mean squared error loss. The blood pressure model was trained with 2 prediction heads as a multitask model for both systolic and diastolic blood pressure. All trained models will be made available through the NAKO TransferHub and are available from the authors with a valid NAKO application.

For the development of deep learning models, we used images that were graded as “good” by the FIT.^8^ We always selected the highest-quality image available for each participant per eye and field. This resulted in a total of 153 134 images from 45 247 participants. The images were resized to 224 × 224 pixels and split into training set (90%) and test set (10%), ensuring that all images from a given participant appeared only in one split. All models were trained using fivefold cross-validation on the training split, with one of the folds serving as the validation set. We selected the best model based on validation performance metrics and then evaluated it on the test set using a bootstrap procedure with 1000 resamples of 15 000 images from the test set. We report the average test set performance and the 95% confidence intervals (CIs), computed as the 2.5 and 97.5 percentiles for various metrics, including accuracy and area under the receiver operating characteristic curve for sex and mean absolute error (MAE) and the coefficient of determination (R^2^) for age, systolic blood pressure, and diastolic blood pressure.

## Results

### Population Profile

The data set consisted of 48 460 participants who had taken part in the baseline ophthalmology module and had ≥1 fundus image for ≥1 eye and a visual acuity examination, as provided by the NAKO TransferHub (Fig 1). Of these, 25 534 were male (52.7%) and 22 926 were female (47.3%) (Table 1). Thus, the percentage of women receiving the baseline eye examination was lower than the percentage of women in the overall NAKO study (50.5%).^22^ The mean age of the participants was 48.9 ± 12.5 years, ranging from 19 to 74 years old, similar to the overall NAKO average (49.9 ± 12.8 years). The different age ranges were well represented: 10.3% of participants were between 20 and 29 years old; 11.3% were between 30 to 39 years old; 27.8% were between 40 and 49 years old; 27.4% were between 50 and 59 years old; 21.5% were between 60 and 69 years old; and 1.7% of participants were between 70 and 79 years old (Supplementary Table 1, available at www.ophthalmologyscience.org). This made the cohort comparatively young at the start of the study.^1^^,^^3^ Participants from all study centers completed the ophthalmic assessment (Supplementary Table 2, available at www.ophthalmologyscience.org).

The cardiovascular and metabolic profile of the participants was typical of a comparatively young adult population cohort (see Table 1; Supplementary Tables 3 and 4, available at www.ophthalmologyscience.org): The mean systolic blood pressure was 127.1 ± 16.2 mmHg (mean ± standard deviation, range 75–236) and the mean diastolic blood pressure was 78.4 ± 10.0 mmHg (range 44–136), with slightly higher systolic blood pressure in men than in women. Based on self-reports, 23.9% of participants reported high blood pressure, while 76.0% reported no history of hypertension. Additionally, 19.4% reported to be active smokers, and 32.6% had smoked in the past, with fewer past smokers in women than in men. In total, 4.9% of participants reported to have diabetes mellitus, with less women than men.

The participants reported a variety of ocular diseases, including cataracts (4.2%), glaucoma (2.0%), and macular degeneration (0.9%) (Table 1). Visual acuity, assessed using logarithm of the minimum angle of resolution measurements, was on average 0.01 ± 0.20 in the left eye (mean ± standard deviation) and 0.03 ± 0.21 in the right eye, with ranges from –0.20 to 1.20 in both eyes, and similar values for men and women. Of the 48 460 participants, 203 493 fundus images were taken, 51 140 from the left central field, 51 112 from the left nasal field, 50 348 from the right central field, and 50 893 from the right nasal field. As per instruction in the standard operating procedures, images were retaken 1 to 2 times for some participants, if the first image was judged to be of insufficient quality; we included all these images in the data set.

### Quality of Fundus Images

We found that the acquired color fundus images were generally of high quality. However, there were also some images of insufficient quality for grading (Fig 2). To systematically evaluate the quality of the acquired fundus images and its dependency on age, we automatically analyzed all 203 493 fundus images of the 48 460 participants with 4 deep learning models for fundus image quality assessment (see Methods). We validated the automatic quality gradings on a subset of 100 images using 5 expert annotators. We found that the average expert quality grades correlated highly with the quality assessment by the deep learning models (Spearman ρ = 0.89/0.88/0.80/0.54 for FIT, AutoMorph, VascX, and RetiRatey, respectively; Supplementary Table 5, available at www.ophthalmologyscience.org). Similar results were also obtained for majority voting for aggregating expert ratings as well as using Cohen κ between binarized model outputs and gradings.

**Figure 2:**
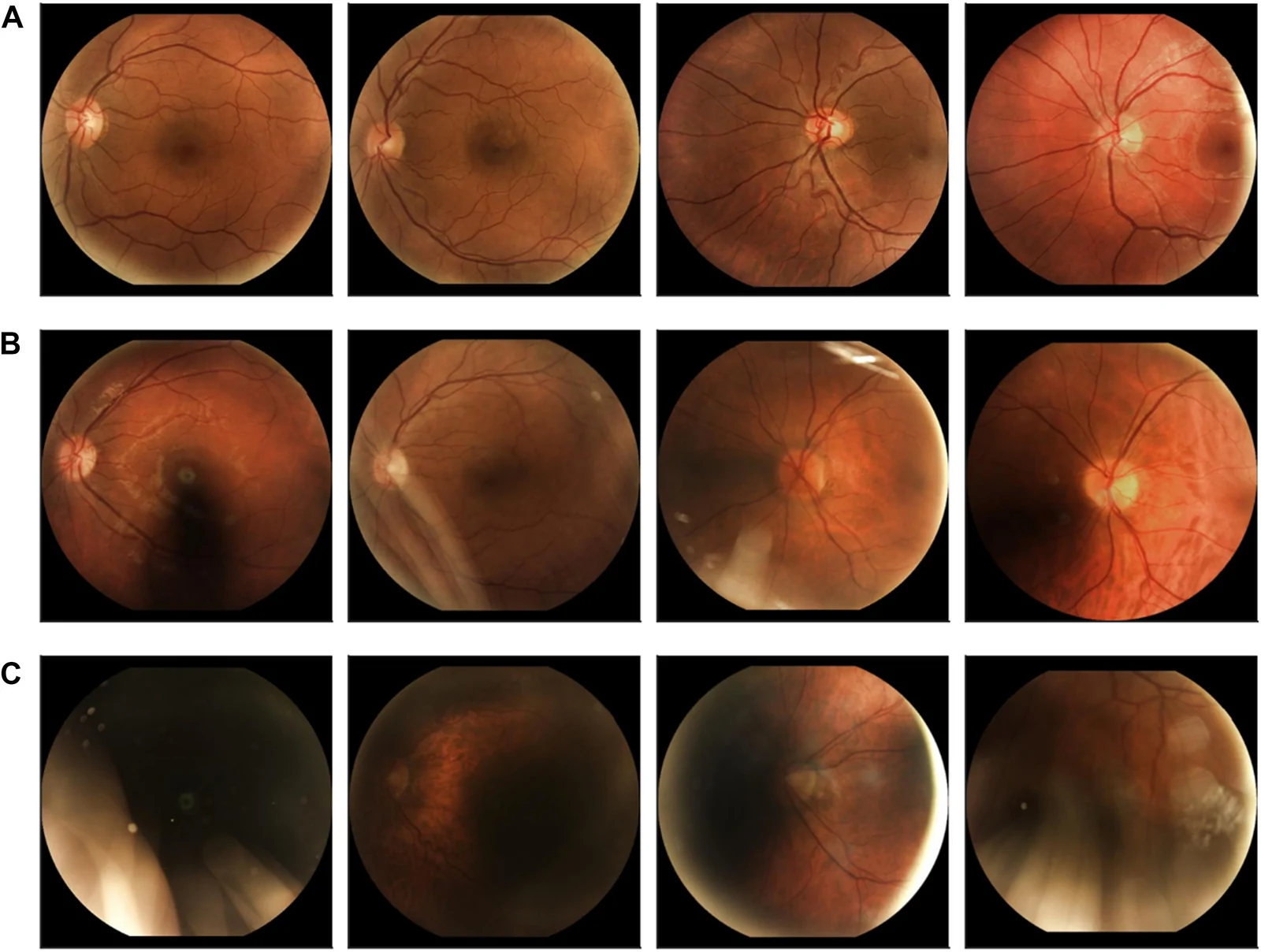
Example images from the NAKO data set. **A,** Fundus images with good quality, as judged by automatic quality grading by the Fundus Image Toolbox algorithm. From left to right: female, 47 years; male, 46 years; male, 32 years; and male, 27 years (as reported during the examination). **B,** Fundus images with borderline quality, showing evidence of artefacts or poor illumination. From left to right: female, 30 years; female, 46 years; female, 73 years; male, 48 years. **C,** Fundus images with insufficient quality not used for further analysis. From left to right: male, 53 years; male, 64 years; male, 63 years; and male, 54 years. NAKO = German National Cohort.

Overall, the 4 algorithms, FIT, RetiRatey, AutoMorph, and VascX, graded 155 857 (76.6%), 177 172 (87.1%), 154 542 (75.9%), and 151 415 (74.4%) out of 203 493 images as gradable, and for 138 885 out of 203 493 (68.2%), all 4 algorithms agreed that these images were of high quality. This percentage is higher than that of other population-based cohort studies, such as the ophthalmic cohort of the UKB (high quality in agreement of all 4 algorithms: 48.7%)^23^ assessed by us using the same algorithms and settings. Depending on the algorithm and the fundus image field, we found that between 70.1% and 91.4% of participants had ≥1 image for ≥1 eye that was classified as good quality (Table 2). Interestingly, the proportion of gradable images was typically higher for the central fields in both eyes, and image quality correlated with the order in which images were taken (right central, right nasal, left central, and left nasal). Image quality declined with increasing age (from 80% to 90% gradable images below 40 years to 40.8% at oldest age group; Supplementary Table 6, available at www.ophthalmologyscience.org) similarly in men and women.

**Table 2:** Percentage of Participants (n = 48 460) with Gradable Images According to 4 Different Artificial Intelligence Models for Quality Grading (FIT, RetiRatey, AutoMorph, and VascX) and Consensus Rating for Participants with Gradable Images According to all Models (All)

Field	FIT	RetiRatey	AutoMorph	VascX	All
Right central	83.1	90.4	85.4	87.7	81.3
Right nasal	83.6	91.4	80.3	72.3	68.2
Left central	76.7	84.7	77.9	82.3	74.9
Left nasal	72.6	89.2	70.1	64.9	58.5

FIT = Fundus Image Toolbox.

### Epidemiological Analysis of Basic Morphological Fundus Properties

To demonstrate the potential of the data set for epidemiological analysis, we used an automatic, externally validated deep learning tool^11^ to extract standardized morphological measurements related to the optic nerve and microvascular health of the fundus, such as the vertical cup-to-disc ratio, CRAE, AVR, and vessel density (see Methods). On average, vertical cup-to-disk ratio was 0.501, CRAE was 154 μm, AVR was 0.679, and vessel density was 0.00689. We examined age- and sex-related differences in retinal and optic nerve head measures. Cup-to-disc ratio increased with age, whereas CRAE, AVR, and vessel density declined from early adulthood (Fig 3). Sex differences were statistically significant but small in magnitude: women showed slightly higher AVR and vessel density and lower cup-to-disc ratios at younger ages, with age-related differences largely similar between sexes and convergence most evident for cup-to-disc ratio at older ages (see Supplementary Tables 7–10, available at www.ophthalmologyscience.org).

**Figure 3:**
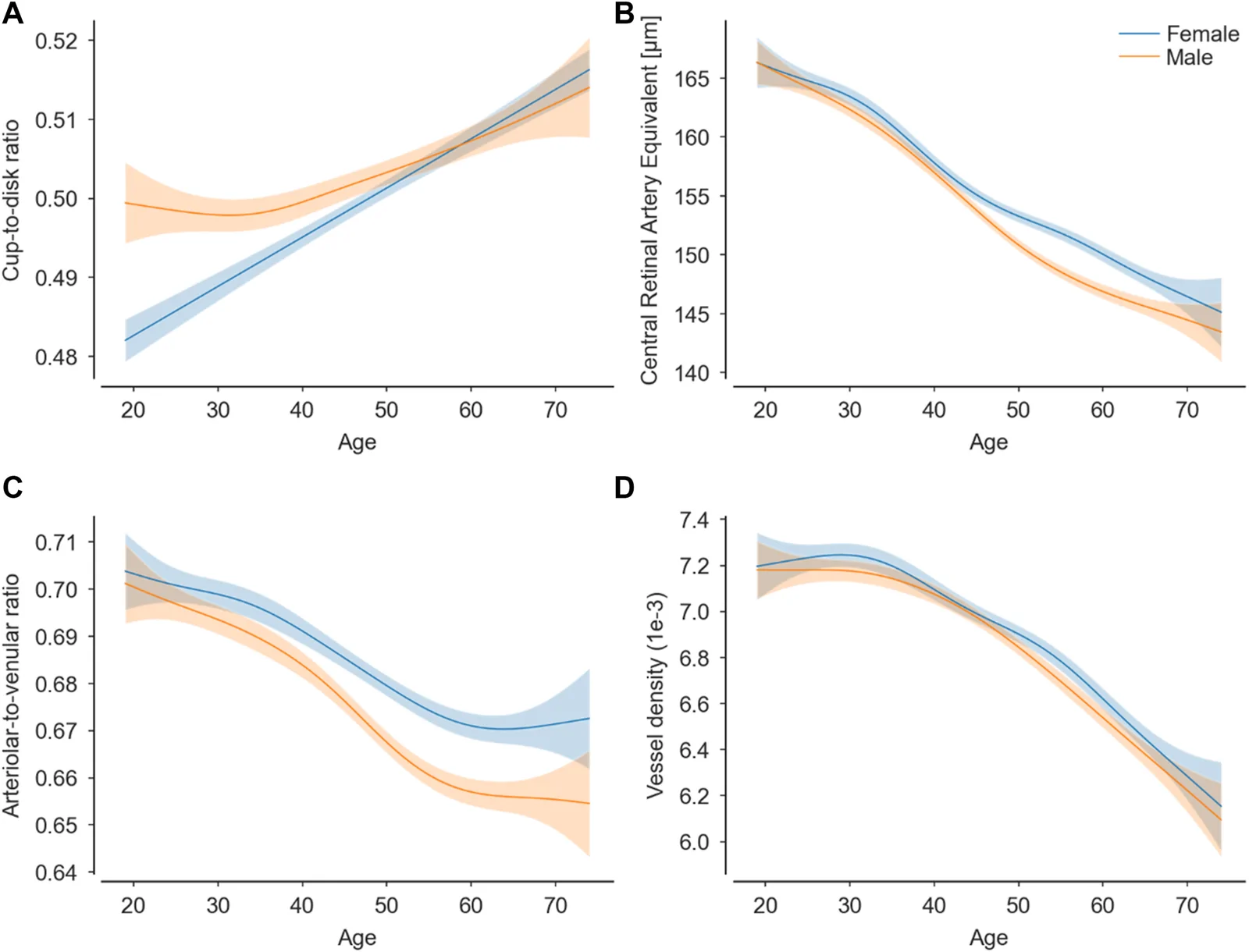
Morphological features extracted from fundus images show dependence on life course and sex. **A,** Cup-to-disc ratio. Solid lines show mean prediction of a fitted generalized additive model, with standard errors. n = 28 220. **B,** Central retinal artery equivalent Knudtson. n = 27 297. **C,** Arteriolar-to-venular ratio Knudtson. n = 27 114. **D,** Vessel density. n = 28 608. Detailed statistical information about the fitted generalized additive model models is available in the Supplementary Material.

### Deep Learning Models for Age, Sex, and Blood Pressure Prediction

To demonstrate the data set’s potential for AI-based ophthalmology and oculomics research, we trained various deep learning models including convolutional and transformer architectures to predict age, sex, and blood pressure from the fundus images based on the data recorded during the baseline examination. These models included ResNet18, ResNet50, Inceptionv3, Xception, EfficientNetv2-Small, ViT-Small, and Swin-Tiny. We found that all architectures could predict these variables with similar performance compared to the literature^24^ (Table 3 and Table 4). For instance, for age prediction, the MAE ranged from 2.964 years (2.920–3.005 95% CI) for the EfficientNet to 3.181 years (3.138–3.223) for the Xception network (Fig 4). For systolic blood pressure prediction, MAE ranged from 10.781 mmHg (10.644–10.922, 95% CI) for the EfficientNet to 11.419 mmHg (11.274–11.585, 95% CI) for the Swin transformer, indicating substantial unexplained variability. The EfficentNetv2 network and the ResNet50 typically achieved the lowest error (Tables 3 and 4), making them good starting points for oculomics approaches.

**Table 3:** Performance of Different Deep Learning Architectures for Prediction of Continuous Variables (Age, Systolic Blood Pressure, and Diastolic Blood Pressure), Measured by the MAE and r^2^, with 95% Confidence Intervals

Target	Model	MAE	R^2^
Age	Resnet18	3.104 (3.063–3.148)	0.889 (0.885–0.893)
	Resnet50	3.079 (3.037–3.121)	0.893 (0.889–0.896)
	Inceptionv3	3.113 (3.074–3.154)	0.889 (0.885–0.893)
	Xception	3.181 (3.138–3.223)	0.885 (0.881–0.889)
	EfficientNetv2-S	2.964 (2.920–3.005)	0.901 (0.898–0.904)
	ViT-Small	3.101 (3.057–3.144)	0.890 (0.886–0.894)
	Swin-Tiny	3.079 (3.038–3.121)	0.894 (0.891–0.898)
	Poplin et al (Inceptionv3)	3.26 (3.22,3.31)	0.74 (0.73,0.75)
Systolic blood pressure	Resnet18	10.923 (10.789–11.052)	0.210 (0.196–0.223)
	Resnet50	10.853 (10.711–10.995)	0.210 (0.196–0.224)
	Inceptionv3	10.950 (10.807–11.101)	0.199 (0.186–0.213)
	Xception	10.906 (10.764–11.049)	0.188 (0.174–0.202)
	EfficientNetv2-S	10.781 (10.644–10.922)	0.215 (0.201–0.229)
	ViT-Small	10.970 (10.829–11.109)	0.200 (0.187–0.212)
	Swin-Tiny	11.419 (11.274–11.585)	0.097 (0.083–0.111)
	Poplin et al (Inceptionv3)	11.35 (11.18,11.51)	0.36 (0.35,0.37)
Diastolic blood pressure	Resnet18	6.969 (6.890–7.051)	0.204 (0.190–0.218)
	Resnet50	6.970 (6.885–7.051)	0.205 (0.192–0.217)
	Inceptionv3	7.090 (7.006–7.171)	0.180 (0.167–0.193)
	Xception	7.067 (6.976–7.152)	0.186 (0.171–0.199)
	EfficientNetv2-S	7.014 (6.931–7.099)	0.201 (0.187–0.214)
	ViT-Small	6.967 (6.888–7.049)	0.204 (0.191–0.217)
	Swin-Tiny	7.242 (7.155–7.327)	0.130 (0.117–0.143)
	Poplin et al (Inceptionv3)	6.42 (6.33, 6.52)	0.32 (0.30, 0.33)

MAE = mean absolute error.

For comparison, literature values from a model trained by Poplin et al on the UK Biobank and EyePACS and evaluated on a holdout set from the UK Biobank.

**Table 4:** Performance of Different Deep Learning Architectures for Prediction of the Binary Variable Sex, Measured by the Accuracy and AUC, with 95% Confidence Intervals

Target	Model	Accuracy	AUC
Sex	Resnet18	0.824 (0.818–0.830)	0.908 (0.903–0.912)
	Resnet50	0.825 (0.818–0.831)	0.910 (0.905–0.914)
	Inceptionv3	0.829 (0.823–0.835)	0.912 (0.907–0.917)
	Xception	0.825 (0.819–0.831)	0.907 (0.903–0.912)
	EfficientNetv2-S	0.835 (0.829–0.841)	0.918 (0.914–0.922)
	ViT-Small	0.820 (0.814–0.826)	0.903 (0.899–0.908)
	Swin-Tiny	0.836 (0.830–0.842)	0.919 (0.915–0.923)
	Poplin et al (Inceptionv3)	-	0.97 (0.966, 0.971)

AUC = area under the receiver operating characteristic curve.

For comparison, literature values from Poplin et al, trained on the UK Biobank and EyePACS and evaluated on a holdout set from the UK Biobank.

**Figure 4:**
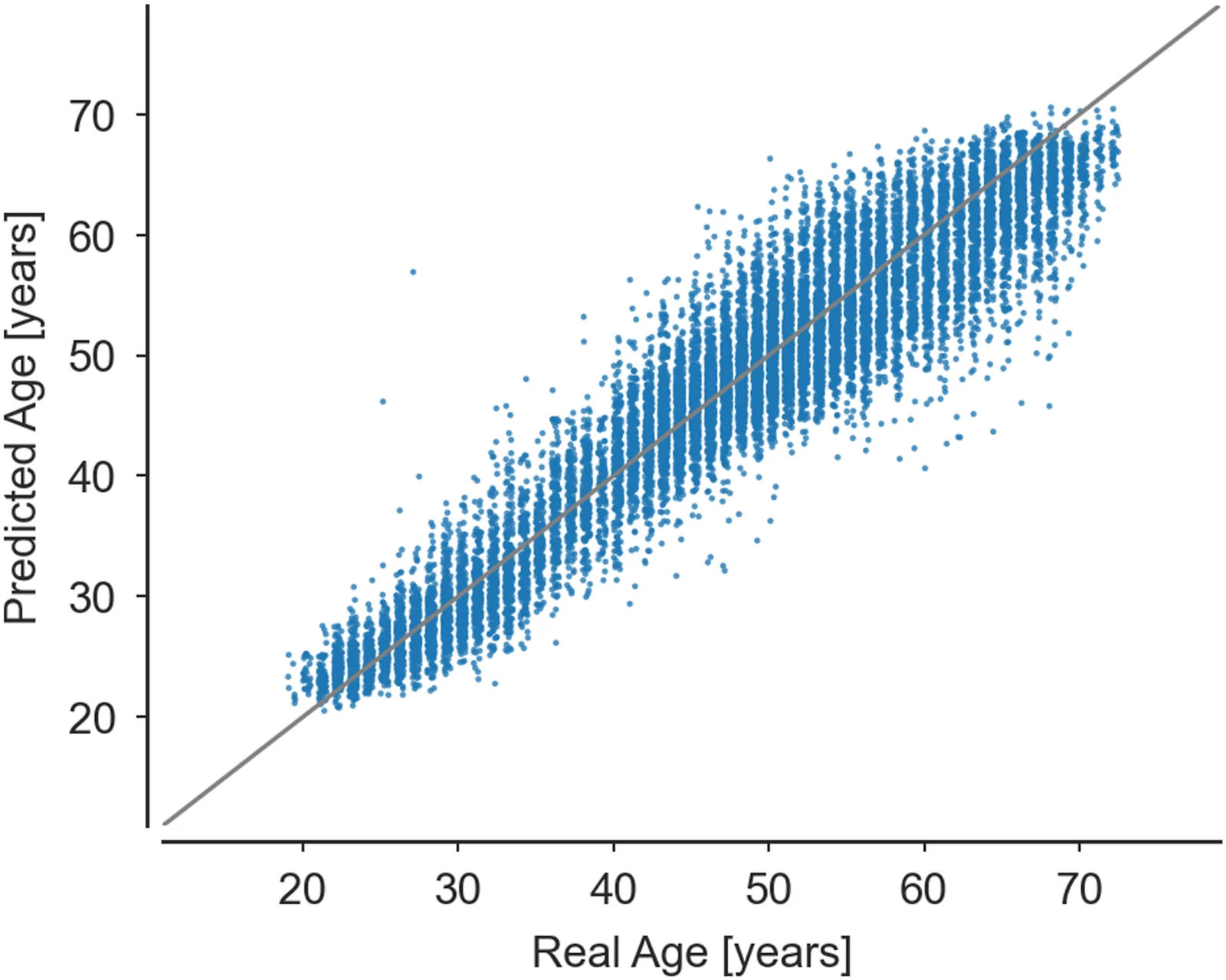
Real vs. predicted age for n = 15 245 NAKO participants for an EfficientNetv2-S model. r^2^ = 0.901, MAE = 2.965. MAE = mean absolute error; NAKO = German National Cohort.

## Discussion

We characterized the design and methodological framework of the ophthalmology module of the NAKO^5^ and present baseline results from 48 460 participants who underwent standardized visual acuity testing and nonmydriatic color fundus imaging, as well as extensive biomedical examination. With its scale, NAKO is among the major international population-based cohorts in ophthalmology such as the UKB baseline ophthalmology subset with around 68 000 participants with OCT and color fundus imaging.^23^^,^^25^ Within Germany, the study complements more local, well-established cohorts in Germany with extensive phenotyping and ophthalmic assessments, such as the Gutenberg Health Study,^26^^,^^27^ the AugUR study,^28^^,^^29^ the Hamburg City Health Study,^30^^,^^31^ the LIFE-Adult Study,^32^ the SHIP study,^33^ the Rhineland Study,^34^, ^35^, ^36^ and the KORA study^37^^,^^38^ (see Table 5 for systematic comparison). In addition to the level 2 ophthalmology module, the NAKO includes local in-depth level 3 modules with ophthalmic relevance (such as for dry eye disease) in selected study centers.

**Table 5:** Comparison of the NAKO Ophthalmology Module with the UK Biobank and Regional German Population-Based Cohorts, Summarizing for Each Cohort Its Geographic Scope, Baseline Sample Size, Age Range, and the Ophthalmic Modalities Assessed (Visual Acuity, Refraction, Color Fundus Photography, OCT, and Additional Modalities)

Cohort	Scope	Sample (Baseline)	Age	VA	Refraction	CFP	OCT	Additional Modalities	Reference
NAKO	National	∼49 000	19–74	X		X			Present study
UK Biobank	National	∼67 000∗	40–69	X	X	X	X	X	Chua 2019
GHS	Regional	∼15 000	35–74	X	X	X	X	X	Höhn 2015
HCHS	Regional	∼10 000 (45 000)†	45–74	X	X	X	X	X	Beuse 2025
LIFE-Adult	Regional	∼10 000	40–79			X	X		Loeffler 2015
Rhineland	Regional	∼5000 (20 000)‡	30–95	X	X	X	X	X	Garzone 2023, Garzone 2025, Mauschitz 2019
SHIP	Regional	∼4000	20–79			X			Jürgens 2014
KORA	Regional	∼3000	25–74			X			Brandl 2016
AugUR	Regional	∼3000	≥70	X		X	X	X	Stark 2015, Brandl 2018

AugUR = age-related diseases: understanding genetic and non-genetic influences – a study at the University of Regensburg; CFP = color fundus photography; GHS = Gutenberg Health Study; HCHS = Hamburg City Health Study; KORA = Cooperative Health Research in the Region of Augsburg; LIFE = Leipzig Research Center for Civilization Diseases; NAKO = German National Cohort; SHIP = Study of Health in Pomerania; UK = United Kingdom; VA = visual acuity.

∗ A total of ∼117 000 participants were screened for vision, intraocular pressure, refraction and keratometry, while a subgroup of ∼67 000 underwent eye imaging.

† Ongoing: published analyses based of the first ∼10 000 of a planned 45 000 cohort.

‡ Ongoing: published analyses based of the first ∼5000 of a planned 20 000 cohort.

The mean logarithm of the minimum angle of resolution values, which were close to 0.0, indicate that this community-based sample predominantly has normal vision. These values are broadly consistent with values reported in large European population-based cohorts, particularly among individuals without significant ocular pathology.^23^^,^^26^ These visual acuity assessments in NAKO will become particularly important when follow-up investigations will be able to compare changes over time as individuals age and derive determinants of aging. Similarly, color fundus images at baseline jointly with follow-up imaging will also be key to derive determinants of eye health changes as well as ocular presentations of other diseases. Together with objectively measured eye parameters, self-reported eye disease can provide insights into the awareness of eye diseases in the general population.

Self-reported eye disease can provide insight into disease awareness in the population, although it is likely to underestimate true disease burden, particularly for early or asymptomatic conditions, and should therefore be interpreted with caution.^26^ As (semi-) automated image-based approaches evolve, they may complement basic self-reported disease assessment and resource and time intensive manual imaging grading for prevalence and incidence estimation by enabling more scalable phenotyping in especially larger data sets. Nonetheless, self-reported prevalence of cataract (4.2%) is consistent with prior evidence indicating that cataract prevalence is often in the single digits in younger populations and increases sharply with age, reaching high levels in older adults. Likewise, the prevalence of self-reported glaucoma (2.0%) aligns with population-based estimates reported in large European cohorts, where glaucoma prevalence generally ranges from approximately 1% to 3%, depending on age and diagnostic definition.^39^, ^40^, ^41^ The prevalence of self-reported macular degeneration (0.9%) is low compared with image-based estimates in European cohorts, where age-related macular degeneration (AMD) prevalence increased with age from 3.5% to 17.6% for early AMD and from 0.1% to 9.8% for late AMD, likely because of the comparably young age of the cohort and the occurrence of symptoms in intermediate and late AMD, while patients with early AMD have little to no symptoms.

Although image quality assessment remains a challenge due to the absence of internationally harmonized standards, we showed that automated quality-control tools can efficiently grade the large collections of fundus images in the NAKO data set. We found that the tools used in this study largely agree on image quality and that their ratings also agree well with expert ratings, despite detectable domain shifts between the training data sets of these algorithms and the NAKO data set. Remaining interalgorithm variability points to the need for further validation, transparency, and consensus guidance.

The large number of high-quality color fundus images from NAKO participants is a key strength of the data set and provides an opportunity to develop AI models for eye health research and oculomics approaches. However, commercial applications require separate disclosure and approval, and fundus images may only be used in publications in a manner that precludes reidentification of participants. The high quality of the fundus images also made it possible to use deep learning–based tools for automatically extracting morphological features. This allowed us to analyze morphological differences of the retina at a larger scale to answer epidemiological questions, as illustrated for age and sex. Quality assessments by the AI models will be made available via the NAKO TransferHub to facilitate further research.

We further demonstrated that the NAKO data set is suited for developing deep learning techniques to study eye health, including oculomics approaches. Baseline deep learning models trained for predicting age, sex, and blood pressure performed similarly to the other approaches based on different fundus image data sets.^24^^,^^42^, ^43^, ^44^ For instance, Poplin et al^24^ trained an Inception-v3 model on subsets of the UKB and the EyePACS data set^45^ to predict cardiovascular risk factors, including sex, age, and blood pressure. They obtained an area under the receiver operating characteristic curve of 0.97 for sex classification, MAE of 3.26 for age prediction, MAE of 11.35 for systolic blood pressure prediction, and MAE of 6.42 for diastolic blood pressure prediction, comparable to the aforementioned values we obtained. Interestingly, our experiments indicate that a ResNet18 (11.2 M), with a comparably small number of parameters, is sufficient to achieve state-of-the-art results, suggesting larger models could be overparametrized for these specific tasks.

The age-related increase in cup-to-disc ratio and the decline in retinal vessel calibers and AVR observed in this study are consistent with prior population-based and photographic studies describing structural remodeling of the optic nerve head and retina with aging.^46^^,^^47^ Sex differences were limited and broadly consistent with previous reports.^47^ These findings should be interpreted in light of the use of measurements of morphological properties obtained using the AutoMorph,^11^ which, while scalable and reproducible, require further validation against manual expert segmentation, as manual annotation remains the current reference standard. Also, the lack of correction for axial length differences could affect these results (see below). Automatically measured morphological properties will be made available via the NAKO TransferHub as derived variables from the data.

Beyond the baseline characterization presented here, the ophthalmic data available within NAKO will continue to grow, as data from follow-up examinations will become available. In addition, manual image gradings and imaging-derived phenotypes will be released, thereby expanding the scientific scope of the data. Follow-up analyses will permit the assessment of changes over time, and previously developed and adapted AI-based methods will enable the efficient provision of further derived variables for use in scientific analyses and for identifying determinants of progression of ocular disease or maintenance of ocular health at older age.

### Strengths and Limitations

Key strengths of NAKO are its large scale, nationwide coverage, and longitudinal design. Standardized operating procedures across study centers, together with high-quality nonmydriatic color fundus photography and visual acuity assessment, support robust population-based analyses. These data enable the investigation of spatial differences and temporal changes. While this present study focused on automated image analysis pipelines, future releases of manually graded imaging data will improve disease-specific eye phenotyping and validation of imaging-derived endpoints. Recent advances in oculomics and AI highlight the potential of retinal imaging as a biomarker for ocular and systemic diseases. However, its large-scale application in population-based cohorts remains limited. The extensive phenotyping in NAKO will allow for detecting unique, new associations of color fundus image features with a broad set of diseases and health conditions.

Several limitations should be acknowledged. First, retinal imaging within the NAKO ophthalmic assessment is limited to nonmydriatic color fundus photography without complementary modalities, such as OCT or widefield imaging. This restricts detailed structural in depth and peripheral phenotyping. Reliance on self-reported ocular disease may also result in misclassification and underascertainment.^48^^,^^49^ Second, we could not correct our morphological measurements for magnification differences due to axial length, refractive error, or corneal curvature, because this information was not available in the data set. Ratio-based measurements like AVR are inherently robust to this, but absolute measurements like CRAE, as well as the reported sex-related differences for these measurements, should be interpreted with care. Third, the width of nonimaging ophthalmic assessment was relatively narrow, encompassing only visual acuity. Fourth, ethnicity data are not available for analysis for NAKO data sets, limiting analyses into fairness and bias in this regard.

## Conclusion and Outlook

The ophthalmic assessment within the NAKO provides a large, standardized, prospective, population-based resource integrating retinal imaging, visual acuity, and comprehensive systemic health measures. We demonstrated its potential as a modern, accessible platform for methodological development and validation in eye health research. Future analyses including follow-up data will provide the opportunity to investigate longitudinal developments among NAKO participants and use the results of the ophthalmic assessment in NAKO for early detection of ocular or systemic diseases.

## Declaration of Generative AI and AI-Assisted Technologies in the Writing Process:

During the preparation of this work the author(s) used DeepL Write and Claude Opus 4.8 in order to improve style and grammar. After using this tool/service, the author(s) reviewed and edited the content as needed and take(s) full responsibility for the content of the publication.

## Footnotes

1 https://transfer.nako.de/transfer/media/NAKO_Data_Handbook_2026_05.pdf.
