# Supplementary material for: The German National Cohort: Ophthalmic Assessment, Baseline Profile, and Potential for Artificial Intelligence–Based Eye Research
Source: Ophthalmol Sci. 2026 Sep 11;6(11):101408. doi: 10.1016/j.xops.2026.101408 (PMC13677226; doi:10.1016/j.xops.2026.101408)
Supplement: Supplementary file 1 [file mmc1.docx]

Supplementary Material for:
The German National Cohort: Ophthalmological Assessment, Baseline Profile and Potential for AI-based Eye Research

Camila Roa ^1,2,*^, Ansgar Beuse ^3,*^, Alexandra Schweig ^4^, Sarah Müller ^1,2^, Klaus Berger^5^, Caroline Brandl^6,7^, Titus Brinker^8^, Anne Elbrecht^9^, Robert Finger^10^, Gerd Geerling^11^, Karin Halina Greiser^8^, Carsten Grohmann^3^, Kathrin Günther^9^, Iris Heid^6^, Andre Karch^5^, Thomas Keil^12, 13, 14^, Jessica Krepel^15^, Michael Leitzmann^16^, Claudia Meinke-Franze^17^, Annette Peters^18, 19^, Sabine Schipf^17^, Matthias Schulz^20,21^, Alexander K. Schuster^22^, Stefan N. Willich^11^, Martin A. Leitritz^4^, Marius Ueffing ^23,#^, Philipp Berens^1,2,#^

^1^ Hertie Institute for AI in Brain Health, University of Tübingen, Tübingen, Germany

^2^ Tübingen AI Center, University of Tübingen, Tübingen, Germany

^3^ Department of Ophthalmology, University Medical Center Hamburg-Eppendorf (UKE), Hamburg, Germany

^4^ University Eye Hospital, Centre for Ophthalmology, University of Tübingen, Tübingen, Germany

^5^ Institute of Epidemiology and Social Medicine, University of Münster, Münster, Germany

^6^ Department of Genetic Epidemiology, University of Regensburg, Regensburg, Germany

^7^ Department of Ophthalmology, University of Regensburg, Regensburg, Germany

^8^ German Cancer Research Center (DKFZ) Heidelberg, Division of Cancer Epidemiology, Heidelberg, Germany

^9^ Leibniz Institute for Prevention Research and Epidemiology – BIPS, Bremen, Germany

^10^ Department of Ophthalmology, Medical Faculty Mannheim, University of Heidelberg, Mannheim, Germany

^11^ Department of Ophthalmology, Heinrich-Heine University, Düsseldorf, Germany

^12^ Institute of Social Medicine, Epidemiology and Health Economics, Charité - Universitätsmedizin Berlin, Berlin, Germany.

^13^ Institute of Clinical Epidemiology and Biometry, University of Würzburg, Würzburg, Germany.

^14^ State Institute of Health I, Bavarian Health and Food Safety Authority, Erlangen, Germany.

^15^ Helmholtz Center for Infection Research, Braunschweig, Germany

^16^ Institute foe Epidemiology und Prevention Medicine, University of Regensburg, Regensburg, Germany

^17^ Institut für Community Medicine, Universitätsmedizin Greifswald, Greifswald, Germany

^18^ Chair of Epidemiology, IBE, Faculty of Medicine, LMU Munich, Germany

^19^ Helmholtz Zentrum München, German Research Center for Environmental Health (GmbH), Institute of Epidemiology, Neuherberg, Germany

^20^ Department of Molecular Epidemiology, German Institute of Human Nutrition Potsdam-Rehbruecke, Nuthetal, Germany

^21^ Institute of Nutritional Science, University of Potsdam, Nuthetal, Germany

^22^ Department of Ophthalmology, University Medical Center of the Johannes Gutenberg-University Mainz, Germany

^23^ Institute for Ophthalmic Research, Centre for Ophthalmology, University of Tübingen, Tübingen, Germany

^*^ Shared first authors. ^#^ Co-senior authors ([marius.ueffing@uni-tuebingen.de](mailto:marius.ueffing@uni-tuebingen.de), [philipp.berens@uni-tuebingen.de](mailto:philipp.berens@uni-tuebingen.de))

Supplementary Table 1: Age distribution of the participants of the baseline from the NAKO Ophthalmology dataset.

| **Age range (years)** | **Male, n (%)** | **Female, n (%)** | **Total, n (%)** |
| --- | --- | --- | --- |
| 10 - 19 | 2 (0.00%) | 2 (0.00%) | 4 (0.01%) |
| 20 - 29 | 2636 (5.44%) | 2343 (4.83%) | 4979 (10.27%) |
| 30 - 39 | 2977 (6.14%) | 2494 (5.15%) | 5471 (11.29%) |
| 40 - 49 | 7136 (14.73%) | 6338 (13.08%) | 13474 (27.80%) |
| 50 - 59 | 6884 (14.21%) | 6394 (13.19%) | 13278 (27.40%) |
| 60 - 69 | 5439 (11.22%) | 4972 (10.26%) | 10411 (21.48%) |
| 70 - 79 | 460 (0.95%) | 383 (0.79%) | 843 (1.74%) |
| All ages | 25534 (52.69%) | 22926 (47.31%) | 48460 (100.00%) |

Supplementary Table 2: Recruitment by study site (n = 48,460).

| **Site** | **n** | **%** |  | **Site** | **n** | **%** |
| --- | --- | --- | --- | --- | --- | --- |
| Augsburg | 8,694 | 17.94 |  | Berlin Nord | 1,618 | 3.34 |
| Neubrandenburg | 5,773 | 11.91 |  | Düsseldorf | 1,609 | 3.32 |
| Mannheim | 4,888 | 10.09 |  | Saarbrücken | 1,586 | 3.27 |
| Essen | 4,373 | 9.02 |  | Regensburg | 1,572 | 3.24 |
| Bremen | 2,067 | 4.27 |  | Hannover | 1,556 | 3.21 |
| Leipzig | 1,933 | 3.99 |  | Kiel | 1,238 | 2.55 |
| Halle | 1,924 | 3.97 |  | Hamburg | 1,211 | 2.50 |
| Münster | 1,917 | 3.96 |  | Neustrelitz | 432 | 0.89 |
| Freiburg | 1,913 | 3.95 |  | Demmin | 415 | 0.86 |
| Berlin Mitte | 1,829 | 3.77 |  | Waren (Müritz) | 89 | 0.18 |
| Berlin Süd | 1,823 | 3.76 |  |  |  |  |

Supplementary Table 3: Summary of demographic, systemic, and eye health variables of n=48,460 participants with complete ophthalmological assessment from the baseline in the NAKO Ophthalmology dataset, split by sex. Continuous variables are reported as mean ± sd and discrete variables as n (%). Note: Percentages are relative to the totals in each column.

| **Variable** | | **Male** | **Female** |
| --- | --- | --- | --- |
| Age (years) | | 48.79 ± 12.58 | 49.05 ± 12.48 |
| Systolic Blood Pressure (mmHg) | | 131.03 ± 14.90 | 122.81 ± 16.40 |
| Diastolic Blood Pressure (mmHg) | | 80.09 ± 9.86 | 76.51 ± 9.74 |
| Left eye logMAR | | 0.00 ± 0.21 | 0.02 ± 0.20 |
| Right eye logMAR | | 0.02 ± 0.21 | 0.04 ± 0.20 |
| Smoking status | Not a smoker Former smoker Smoker Missing | 11029 (43.19%) 8797 (34.45%) 5155 (20.19%) 553 (2.17%) | 11807 (51.50%) 6506 (28.38%) 4030 (17.58%) 583 (2.54%) |
| High Blood Pressure | No Yes Missing | 18049 (70.69%) 7467 (29.24%) 18 (0.07%) | 18803 (82.02%) 4099 (17.88%) 24 (0.10%) |
| Diabetes Mellitus | No Yes Missing | 24150 (94.58%) 1349 (5.28%) 35 (0.14%) | 21866 (95.38%) 1031 (4.50%) 29 (0.13%) |
| Cataracts | No Yes Missing | 24541 (96.11%) 926 (3.63%) 67 (0.26%) | 21756 (94.90%) 1116 (4.87%) 54 (0.24%) |
| Glaucoma | No Yes Missing | 25007 (97.94%) 446 (1.75%) 81 (0.32%) | 22331 (97.40%) 525 (2.29%) 70 (0.31%) |
| Macular Degeneration | No Yes Missing | 25225 (98.79%) 223 (0.87%) 86 (0.34%) | 22645 (98.77%) 206 (0.90%) 75 (0.33%) |

Supplementary Table 4: Summary of demographic, systemic, and eye health variables of n=48,460 participants with complete ophthalmological assessment from the baseline in the NAKO Ophthalmology dataset, split by age group. Continuous variables are reported as mean ± sd and discrete variables as n (%). Note: Percentages are relative to the totals in each column.

| **Variable** | | **Age range (years)** | | |
| --- | --- | --- | --- | --- |
|  |  | **<40** | **40 - 60** | **> 60** |
| Systolic Blood Pressure (mmHg) | | 120.89 ± 12.41 | 126.44 ± 15.47 | 134.6 ± 17.87 |
| Diastolic Blood Pressure (mmHg) | | 73.99 ± 8.59 | 79.7 ± 9.97 | 79.39 ± 9.98 |
| Left eye logMAR | | -0.06 ± 0.16 | 0.00 ± 0.20 | 0.09 ± 0.21 |
| Right eye logMAR | | -0.05 ± 0.17 | 0.02 ± 0.20 | 0.11 ± 0.22 |
| Sex | Male Female | 5615 (53.71%) 4839 (46.29%) | 14020 (52.41%) 12732 (47.59%) | 5899 (52.42%) 5355 (47.58%) |
| Smoking status | Not a smoker Former smoker Smoker Missing | 5755 (55.05%) 2230 (21.33%) 2338 (22.36%) 131 (1.25%) | 12286 (45.93%) 8524 (31.86%) 5419 (20.26%) 523 (1.95%) | 4795 (42.61%) 4549 (40.42%) 1428 (12.69%) 482 (4.28%) |
| High Blood Pressure | No Yes Missing | 9449 (90.39%) 996 (9.53%) 9 (0.09%) | 20472 (76.53%) 6257 (23.39%) 23 (0.09%) | 6931 (61.59%) 4313 (38.32%) 10 (0.09%) |
| Diabetes Mellitus | No Yes Missing | 10297 (98.50%) 149 (1.43%) 8 (0.08%) | 25690 (96.03%) 1030 (3.85%) 32 (0.12%) | 10029 (89.11%) 1201 (10.67%) 24 (0.21%) |
| Cataracts | No Yes Missing | 10428 (99.75%) 17 (0.16%) 9 (0.09%) | 26219 (98.01%) 491 (1.84%) 42 (0.16%) | 9650 (85.75%) 1534 (13.63%) 70 (0.62%) |
| Glaucoma | No Yes Missing | 10408 (99.56%) 29 (0.28%) 17 (0.16%) | 26242 (98.09%) 442 (1.65%) 68 (0.25%) | 10688 (94.97%) 500 (4.44%) 66 (0.59%) |
| Macular Degeneration | No Yes Missing | 10423 (99.70%) 16 (0.15%) 15 (0.14%) | 26497 (99.05%) 177 (0.66%) 78 (0.29%) | 10950 (97.30%) 236 (2.10%) 68 (0.60%) |

Supplementary Table 5: Agreement between the quality rating of five expert annotators on 100 fundus images and the model output. Left column: Spearman’s rho, measuring the rank agreement between the continuous model output and the average or majority expert annotation. Right column: Cohen’s kappa, measuring inter-annotator agreement on binarized model outputs and ratings.

|  | **Spearman’s rho** | | **Cohen's Kappa  (binary)** | |
| --- | --- | --- | --- | --- |
| **Model** | **Average** | **Majority** | **Average** | **Majority** |
| FIT | 0.89 | 0.84 | 0.74 | 0.71 |
| AutoMorph | 0.88 | 0.85 | 0.86 | 0.83 |
| VascX | 0.8 | 0.76 | 0.72 | 0.68 |
| RetiRatey | 0.54 | 0.54 | 0.68 | 0.64 |

Supplementary Table 6: Percentage of good quality images from participants of each age range (n=203,493). Percentages are relative to the total number of available images per age range.

| **Age range (years)** | **FIT** | **RetiRatey** | **AutoMorph** | **VascX** | **All** |
| --- | --- | --- | --- | --- | --- |
| 10 - 19 | 93.8 | 93.8 | 93.8 | 93.8 | 93.8 |
| 20 - 29 | 90.2 | 93.6 | 89.5 | 89.3 | 86.2 |
| 30 - 39 | 86.6 | 91.2 | 86.0 | 85.0 | 81.1 |
| 40 - 49 | 82.6 | 89.2 | 81.5 | 79.3 | 74.6 |
| 50 - 59 | 75.5 | 86.4 | 74.6 | 72.1 | 65.6 |
| 60 - 69 | 60.9 | 80.8 | 61.0 | 60.4 | 50.7 |
| 70 - 79 | 50.8 | 76.9 | 50.5 | 52.1 | 40.8 |

Supplementary Table 7: Summary of the GAM fitted to cup-to-disc ratio measurements.

| **A. parametric coefficients** | **Estimate** | **Std. Error** | **t-value** | **p-value** |
| --- | --- | --- | --- | --- |
| (Intercept) | 0.50 | 0.00 | 909.19 | < 0.0001 |
| sex Male | 0.00 | 0.00 | 4.65 | < 0.0001 |
| **B. smooth terms** | **edf** | **Ref.df** | **F-value** | **p-value** |
| s(age): sex Female | 1.01 | 1.02 | 197.52 | < 0.0001 |
| s(age): sex Male | 2.82 | 3.52 | 50.89 | < 0.0001 |
| **C. other stats** | **R2** | **Deviance explained** | **REML** | **n** |
|  | 0.00925 | 0.94% | -36976 | 28220 |

Supplementary Table 8: Summary of the GAM fitted to central retinal artery equivalent (CRAE) measurements.

| **A. parametric coefficients** | **Estimate** | **Std. Error** | **t-value** | **p-value** |
| --- | --- | --- | --- | --- |
| (Intercept) | 155.35 | 0.15 | 1071.63 | < 0.0001 |
| sex Male | -1.89 | 0.20 | -9.27 | < 0.0001 |
| **B. smooth terms** | **edf** | **Ref.df** | **F-value** | **p-value** |
| s(age): sex Female | 4.88 | 5.93 | 1323.27 | < 0.0001 |
| s(age): sex Male | 4.32 | 5.31 | 1794.85 | < 0.0001 |
| **C. other stats** | **R2** | **Deviance explained** | **REML** | **n** |
|  | 0.104 | 10.50% | 115850 | 27297 |

Supplementary Table 9: Summary of the GAM fitted to arteriolar-to-venular ratio measurements.

| **A. parametric coefficients** | **Estimate** | **Std. Error** | **t-value** | **p-value** |
| --- | --- | --- | --- | --- |
| (Intercept) | 0.68 | 0.00 | 1003.38 | < 0.0001 |
| sex Male | -0.01 | 0.00 | -10.53 | < 0.0001 |
| **B. smooth terms** | **edf** | **Ref.df** | **F-value** | **p-value** |
| s(age): sex Female | 3.81 | 4.72 | 238.12 | < 0.0001 |
| s(age): sex Male | 4.15 | 5.12 | 442.26 | < 0.0001 |
| **C. other stats** | **R2** | **Deviance explained** | **REML** | **n** |
|  | 0.0279 | 2.82% | -30337 | 27114 |

Supplementary Table 10: Summary of the GAM fitted to vessel density measurements.

| **A. parametric coefficients** | **Estimate** | **Std. Error** | **t-value** | **p-value** |
| --- | --- | --- | --- | --- |
| (Intercept) | 0.01 | 0.00 | 638.68 | < 0.0001 |
| sex Male | 0.00 | 0.00 | -3.54 | 0.0004 |
| **B. smooth terms** | **edf** | **Ref.df** | **F-value** | **p-value** |
| s(age): sex Female | 4.39 | 5.39 | 571.31 | < 0.0001 |
| s(age): sex Male | 3.71 | 4.61 | 613.21 | < 0.0001 |
| **C. other stats** | **R2** | **Deviance explained** | **REML** | **n** |
|  | 0.0399 | 4.02% | -149620 | 28608 |
